# Synthesis and Characterization of Nanofunctionalized Gelatin Methacrylate Hydrogels

**DOI:** 10.3390/ijms18122675

**Published:** 2017-12-10

**Authors:** Kamel Rahali, Ghazi Ben Messaoud, Cyril J.F. Kahn, Laura Sanchez-Gonzalez, Mouna Kaci, Franck Cleymand, Solenne Fleutot, Michel Linder, Stéphane Desobry, Elmira Arab-Tehrany

**Affiliations:** 1 Laboratoire d’Ingénierie des Biomolécules (LIBio), Université de Lorraine, 2 Avenue de la Forêt de Haye–BP 20163, 54505 Vandoeuvre-lès-Nancy, France; kamel.rahali.iut@gmail.com (K.R.); ghazi.benmessaoud@hotmail.fr (G.B.M.); cyril.kahn@univ-lorraine.fr (C.J.F.K.); laura.sanchez-gonzalez@univ-lorraine.fr (L.S.-G.); mouna.kaci@gmail.com (M.K.); michel.linder@univ-lorraine.fr (M.L.); stephane.desobry@univ-lorraine.fr (S.D.); 2 Institut Jean Lamour (UMR CNRS 7198), Université de Lorraine, Parc de Saurupt, CS 50840, 54011 Nancy CEDEX, France; franck.cleymand@univ-lorraine.fr (F.C.); solenne.fleutot@univ-lorraine.fr (S.F.)

**Keywords:** GelMA, functionalized hydrogel, nanoliposome, nanoemulsion, LF/HF ultrasounds, mechanical properties, tissue engineering

## Abstract

Given the importance of the extracellular medium during tissue formation, it was wise to develop an artificial structure that mimics the extracellular matrix while having improved physico-chemical properties. That is why the choice was focused on gelatin methacryloyl (GelMA), an inexpensive biocompatible hydrogel. Physicochemical and mechanical properties were improved by the incorporation of nanoparticles developed from two innovative fabrication processes: High shear fluid and low frequencies/high frequencies ultrasounds. Both rapeseed nanoliposomes and nanodroplets were successfully incorporated in the GelMA networks during the photo polymerization process. The impact on polymer microstructure was investigated by Fourier-transform infrared spectroscopy (FTIR), scanning electron microscopy (SEM), and enzymatic degradation investigations. Mechanical stability and viscoelastic tests were conducted to demonstrate the beneficial effect of the functionalization on GelMA hydrogels. Adding nanoparticles to GelMA improved the surface properties (porosity), tuned swelling, and degradability properties. In addition, we observed that nanoemulsion didn’t change significantly the mechanical properties to shear and compression solicitations, whereas nanoliposome addition decreased Young’s modulus under compression solicitations. Thus, these ways of functionalization allow controlling the design of the material by choosing the type of nanoparticle (nanoliposome or nanoemulsion) in function of the application.

## 1. Introduction

Hydrogels are polymeric networks made of hydrophilic groups or domains, which allowed the material to absorb and retain large amounts of water. Thus, to avoid dissolution of the hydrogel into the aqueous phase, crosslinks have to be present [1].

Hydrogels were first synthesized by Wichterle and Lίm in 1960 [2]. Since then, they have been developed for a widely range of applications such as food additives, biomedical implants, pharmaceutical & diagnostics products, biosensors, drug carriers, and wound dressing materials [3,4,5,6,7]. Today, hydrogels are extensively studied as biomaterials due to their properties (mechanical, physicochemical, etc.) and functional resemblance with the extracellular matrix (ECM) [1]. Thus, their characteristics and biocompatibility make them an interesting research material in the fields of tissue engineering, cell encapsulation, and drug delivery applications [8].

Hydrogels contain hydrophilic groups that, in an aqueous medium, absorb a large amount of water into its pores and develop a superabsorbent material [9,10,11].

The water retention capacity of these type of materials can be considered as one of their most important characteristic property where they have the ability to imbibe water up to 20 times more than its original molecular weight [12] and becoming soft with a degree of flexibility similar to living tissues [13,14].

Hydrogel scaffolds can be formed by natural and/or synthetic crosslinked polymer chains [15] Synthetic polymers allow a control of their composition, polymerization, and mechanical properties and their microstructure, thereby their degradability. The chemical properties of hydrogels to create an optimized cellular environment that improve the cellular performance and activities [8,16,17,18]. In addition to their physicochemical characteristics, hydrogels can be functionalized by the incorporation of molecules of interest or vectors for the delivery of active molecules in a native functional state.

Among the various hydrogels, gelatin is one of these natural polymers that are extensively used in biomedical application [19,20]. It is a natural cytocompatible protein [21] derived from either an acid or an alkaline hydrolysis of a natural product called collagen [22,23].

It is an interesting polymer due to the presence of many bioactive sequences such as arginine-glycine-aspartic acid (RGD), providing cell attachment and growth of different type of active molecules [24,25,26]. However, to enhance its final physicochemical properties and avoid the thermo reversibility due to its relatively low melting point, a chemical modification of this polymer is necessary. For this, gelatin methacryloyl (GelMA), a semi synthetic derived hydrogel, was chosen [19].

GelMA is produced by the substitution of the free amine groups of the gelatin with methacrylate anhydride while preserving the arginine-glycine-aspartic acid (RGD) sequences that promote cell attachment. Conveniently, introduction of methacryloyl substituent groups and photoinitiator confers to gelatin the property of photocrosslinking by exposure to UV radiation [27]. This polymerization does not need to be done at specific conditions (room temperature, neutral pH, in aqueous environments, etc.), and allows the control of the reaction in terms of time and space [26]. This enables microfabrication of the hydrogels to create unique patterns, morphologies, and 3D structures for applications such as platforms to control cellular behaviors in order to study cell-biomaterial interactions, cell-laden microtissues and microfluidic devices [28].

Properties of GelMA (swelling, strength, porosity, etc.) can be optimized further by incorporating nanoparticles within the hydrogel. Previous works [29,30] showed that nanoliposomes improve mechanical properties and porosity of the GelMA hydrogels. In order to compare the various properties of GelMA functionalized with lipidic nanoparticles differing by their external surface hydrophilicity and their structure. Thus, using the nanoemulsion as soft nanoparticle [31] can be another possibility to improve the physico-chemical properties of GelMA compared to GelMA-Nanoliposome functionalization.

## 2. Results and Discussion

### 2.1. Solutions Characterization

Size, polydispersity index, and zeta potential of nanoliposomes solution and nanoemulsion were measured immediately after preparation (Table 1). The average size of rapeseed nanoliposomes was 170 nm, and it was consistent with the results previously obtained with the sonication method [32] with a better polydispersity index. Rapeseed nanodroplets average size was 238 nm with a polydispersity index extremely close to that of nanoliposomes. This index corresponds to a good distribution of the particles and a good homogeneity of the solution. Zeta potentials were substantially similar with −43.7 mV and −48.8 mV, respectively, for liposomes and oil nanodroplets. It is due to the negative electrophoretic mobility of the phospholipids in nanoliposomes [30,33] and the hydroxide ions in the oil nanodroplets [34]. Small particle size, low polydispersity index, and negative charges are important to prevent an aggregation of the liposomes or a coalescence of the droplets during functionalization of the hydrogels.

### 2.2. Hydrogels Morphology

**Figure 1:**
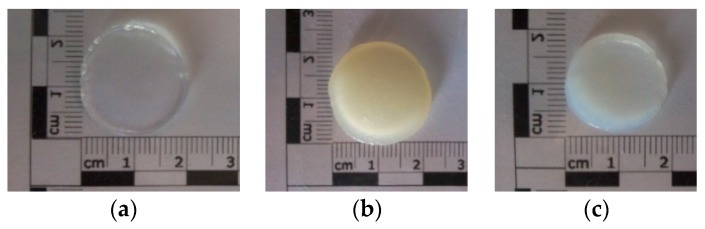
GelMA scaffolds after photopolymerization (**a**) 15% GelMA; (**b**) GelMA-Nanoliposomes 15%:1.5% (*w*/*w*); (**c**) GelMA-Emulsion 15%:1.5% (*w*/*w*).

After photopolymerization of the hydrogels under UV irradiation, the rinsed hydrogel discs had smooth and homogenous surfaces: pure 15% GelMA disc was completely transparent, while functionalized GelMA discs were translucent for GelMA-Nanoemulsion and opaque for GelMA-Nanoliposome. GelMA-Nanoliposomes hydrogel was a yellowish colored opaque and GelMA-Emulsion hydrogel was translucent white. Coloration of the hydrogels indicates that the incorporation of nanoliposomes from rapeseed lecithin and nanodroplets from rapeseed emulsion are uniform (Figure 1).

### 2.3. Fourier-Transform Infrared Spectroscopy (FTIR)

**Figure 2:**
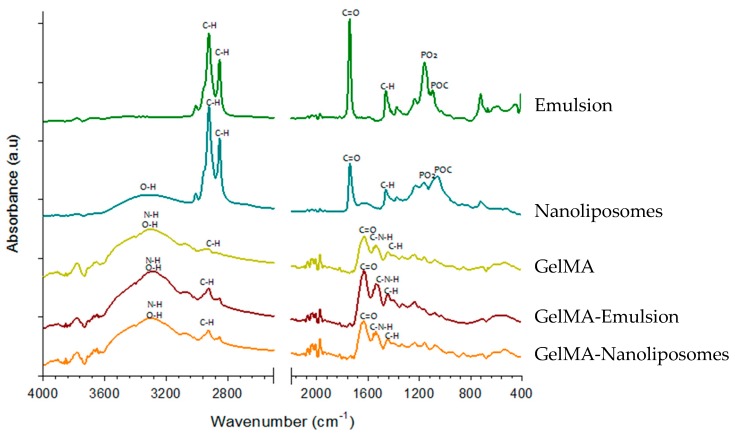
Fourier-transform infrared spectroscopy (FTIR) spectra of Emulsion, Nanoliposomes, GelMA, GelMA-Emulsion, and GelMA-Nanoliposomes. a.u., arbitrary unit.

Material excited by infrared sources can be characterized by the spectra of molecular absorption and transmission peaks obtained from vibration frequencies between the bounds of atoms. Fourier-transform infrared spectroscopy (FTIR) is used essentially to characterize the presence of specific chemical groups in the hydrogels and to study the interaction between the blended polymers and the effect of the added nanoliposomes or nanodroplets in the polymers. The results show the spectrum of nanoliposomes, emulsion, pure GelMA hydrogel, and GelMA functionalized with nanoliposomes or nanoemulsion (Figure 2).

The FTIR spectrum of pure rapeseed oil (emulsion) presents the 22 specific regions [35,36]. It consists of a major quantity of triglycerides that the main bands appearing in spectrum due to the asymmetric and symmetric stretching vibration of CH at 2924 and 2854 cm^−1^ and stretching of C=O at 1744 cm^−1^. Weaker bands appear at 1464, 1377, 1242, 1161, 1119 and 723 cm^−1^ that correspond to scissoring of CH (CH_2_), bending of CH (CH_3_), CH (cis), CH_2_, CO stretching, and rocking of –(CH_2_)*_n_*–, respectively. The spectrum of nanoliposomes displays the main characteristic bands of phospholipids presented in liposomes: maximum of peaks at 2854 and 2924 cm^−1^, corresponding to the symmetric and antisymmetric stretching in the CH_2_ groups of alkyl chains, respectively. The broad band from 3750 to 3050 cm^−1^ represents OH band. The band at 1732 cm^−1^ corresponds to the stretching vibrations of the ester carbonyl groups of phospholipids, and the relatively strong band centered at 1651 cm^−1^ corresponds to the stretching vibrations of alkene carbon–carbon double bond –C=C–. The scissoring vibrations of the CH_2_ groups are represented by the band at 1456 cm^−1^, and the band at 1406 cm^−1^ corresponds to (=C–H) bending (rocking) vibrations. While the relatively weak band at 1394 cm^−1^ represents the umbrella deformation vibrations of the CH_3_ groups of alkyl chains. In addition, the spectral bands at 1086 and 1224 cm^−1^ represent the symmetric and antisymmetric PO_2_– stretching vibration of phospholipids, and the band representing the antisymmetric N^+^/CH_3_ stretching vibrations was detected at 970 cm^−1^ [37].

All the hydrogels spectra (GelMA, GelMA-Emulsion, and GelMA-Nanoliposomes) showed a broad peak with a peak position at 3290 cm^−1^ associated with the stretching of the hydrogen bonded hydroxyl groups. The spectrum of GelMA hydrogel derived from the modification of the gelatin with the methacrylate anhydride. A strong peak appears at 1650 cm^−1^ related to amide I primarily C=O stretching groups. The band at 1500–1570 cm^−1^ corresponds to C–N–H bending while the band at 3200–3400 cm^−1^ indicates the presence of peptide bonds (mainly N–H stretching). The peak at 3062 cm^−1^ represents the C–H stretching groups [38,39]. The peak at 1640 cm^−1^ corresponds to carbon double bond in GelMA that presents the interaction between gelatin and methacrylate anhydride. The spectra of the hydrogel with the soft nanoparticles (GelMA-Nanoliposomes) does not show the peaks corresponding to the liposomes spectra but it presents an increasing of the intensity of certain peaks specially around 3300 cm^−1^. The spectra of hydrogel with emulsion (GelMA-Emulsion) presents a similar increasing in the same region corresponding to the presence of a small amount of fatty acids. These results show an interaction between the GelMA and the nanoliposomes and nanoemulsion.

### 2.4. Scanning Electron Microscopy (SEM)

**Figure 3:**
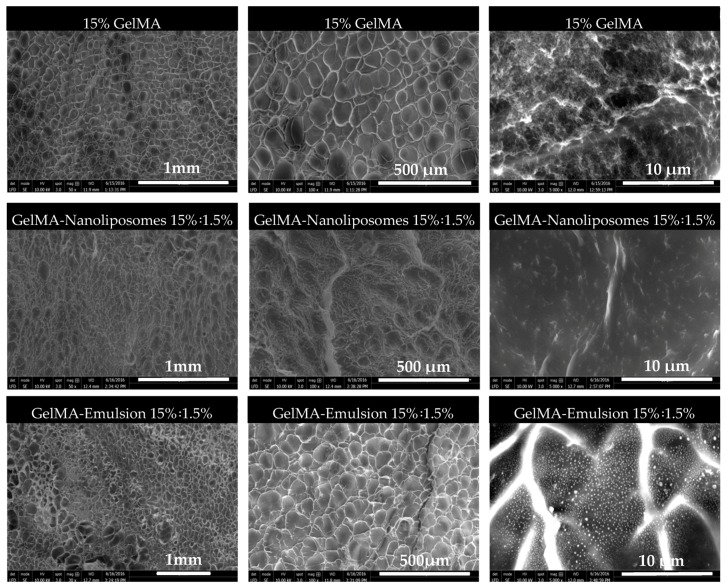
Scanning electron microscopy (SEM) images of 3D hydrogels (GelMA, GelMA-Nanoliposomes, and GelMA-Emulsion).

The morphological properties of pure GelMA and functionalized hydrogels were investigated by scanning electron microscopy (SEM). Upon examination with SEM, we observed the porosity of GelMA hydrogels with different diameters on the microscale (Figure 3).

Although freeze drying procedure can alter material porosity, all samples were dried by the same process and there was no difference in the pore distribution. Pores were shaped pockets like which do not seem interconnected and are separated by thin walls. They are different size sand were formed during the photopolymerization, the syneresis phenomenon acts by squeezing locally excess of water out of the polymer in an attempt to reach its equilibrium swelling concentration (by a thermodynamically favored state of the polymer) and prevent the system from forming a fully homogenous material [40]. Higher magnification (Figure 3, ×5000; scale bar 10 µm) showed that nanoliposomes were totally assimilated (a homogenous surface morphology) in the network, while nanodroplets were adsorbed at the surface of GelMA after nanofunctionalization.

### 2.5. Degradability

For biomedical applications, biodegradability of hydrogel materials must be investigated. Indeed, in order to develop and obtain a mature tissue, cells must be able to degrade and remodel their hydrogel environment. GelMA has already been tested, and like gelatin, in its native state, maintains its susceptibility to enzymatic degradation. Thus, the influence of functionalized by nanoliposome or nanoemulsion hydrogel must investigate toward enzymatic activity. GelMA hydrogel and functionalized hydrogels were added to collagenase type II dissolved in phosphate buffer solution (PBS) at an enzyme concentration of 2 µg/mL. Enzymatic degradation of the GelMA hydrogels was examined to ensure that the inlay of nanoparticles did not decrease the enzymatic degradability.

**Figure 4:**
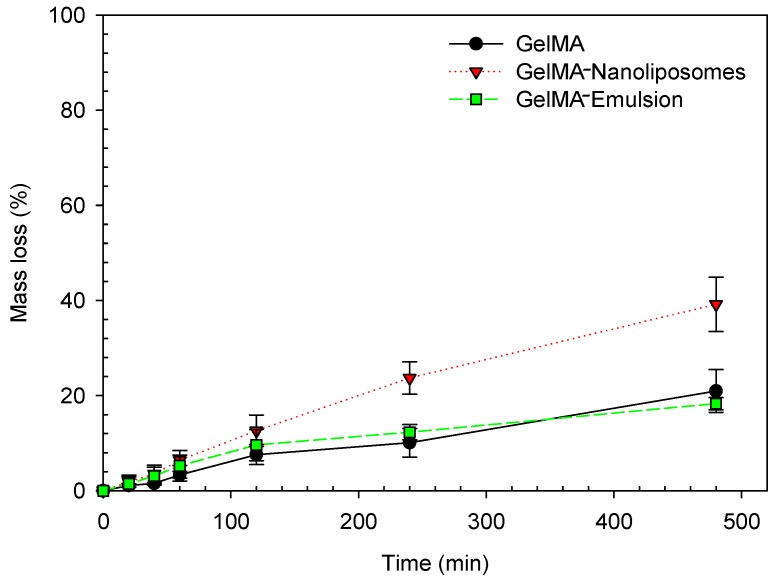
GelMA degradability GelMA (●), GelMA-Nanoliposomes (▼), and GelMA-Emulsion (■) hydrogels of uniform size were exposed to 2 µg/mL exogenous collagenase. Mass losses (%) were measured during 8 h. Error bars represent standard deviation.

The degradation profiles of GelMA, GelMA-Emulsion, and GelMA-Nanoliposomes (Figure 4) showed that at a concentration of 2 µg/mL after 2 h of incubation the degradation rate was the same in the three systems, but after 4 h of incubation, type II collagenase had a tendency to degrade the GelMA-Nanoliposomes most rapidly. This tendency was confirmed after 8 h of incubation, and the rate of mass loss was approximately double (39.2% in GelMA-Nanoliposomes hydrogels against 21.0% and 18.3% in pure GelMA and GelMA-Emulsion hydrogels, respectively). This increase can be due to a physical phenomenon, as nanoliposomes incorporation increases pores size and enhances their distribution [29], which releases a way of privileged access to the collagenase, multiplying by twice the area of access sequences Arg-Gly-Asp (RGD), which are considered matrix metalloproteinase 2 (MMP-2) binding sites. These enzymes can degrade and remodel the extracellular matrix for cell spreading and migration.

### 2.6. Swelling Behavior Study

To study of swelling characteristics of polymers allows us to evidence the surface properties as well as to explain the mechanical characteristics and the diffusion process. The pore size of a polymer network, the interaction between the polymer and the solvent, the methacrylation degree, and the amount of photoinitiator will determine its degree of swelling. That is why we investigated the mass swelling ratio of 15% GelMA polymers (pure and functionalized) in two different solvents (water and PBS solution, pH = 7.4).

**Figure 5:**
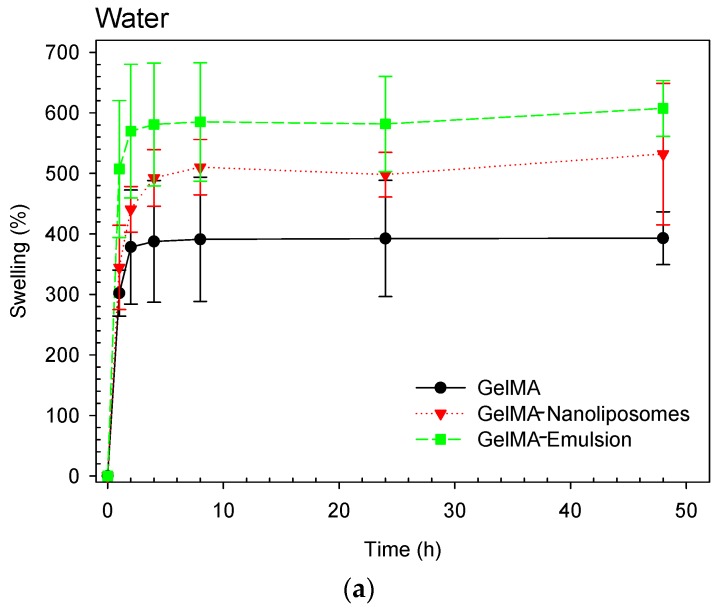

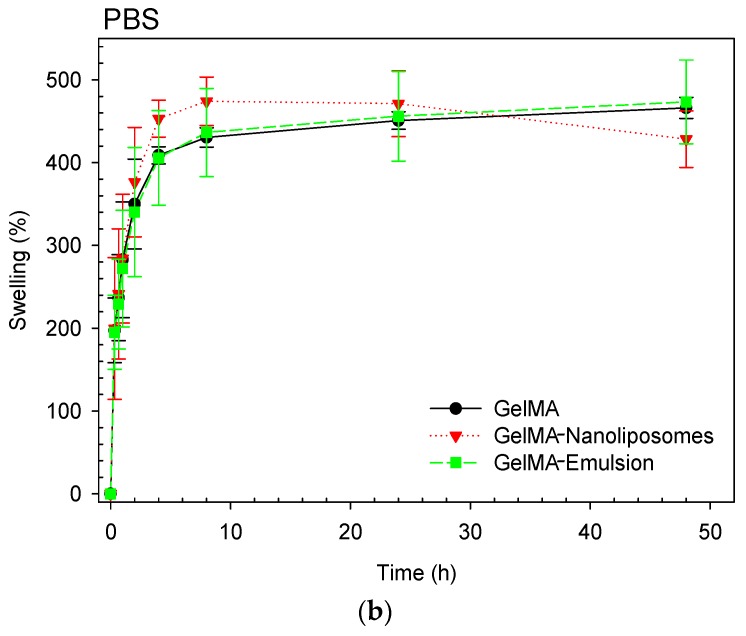
Degree of swelling of based hydrogels (GelMA (●), GelMA-Nanoliposomes (▼), and GelMA-Emulsion (■)) in two different solvents (**a**: deionized water and **b**: PBS solution), at 37 °C. Error bars represent standard deviation.

The calculated swelling ratio in water (Figure 5a) demonstrated that the functionalization of the GelMA did not affect the water retention capacity, and there was no significant difference between the hydrogels, they have absorbed in mean from four to six times their weight of water. In PBS solution (Figure 5b), the three hydrogels swallowed with the same proportions from four to five times their weight of water. However, a difference in the equilibrium time was observed, in deionized water the equilibrium time was reached after 2 h only while otherwise in PBS solution, it took four times longer. This difference can be explained by a capillary process associated with a higher osmosis pressure due to the deionized water which accelerate filling of interconnected pores.

### 2.7. Mechanical Stability

**Figure 6:**
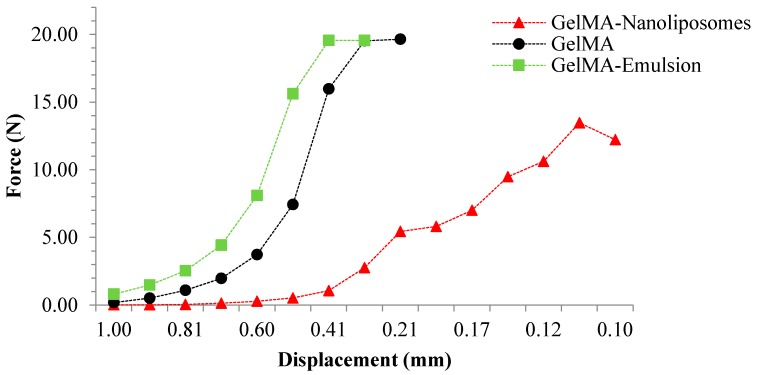
Typical force–displacement curves of GelMA hydrogels: pure GelMA (●) and Functionalized GelMA (▲: GelMA-Nanoliposomes and ■: GelMA-Emulsion).

The compression curves of the hydrogels (Figure 6) showed the same force–displacement behavior for GelMA and GelMA-Emulsion hydrogels. In the case of these two systems, it is observed that the deformation requires a force superior to the limit of the device. The earlier increase can be related to the stiffness of the crosslinked GelMA (Young’s modulus of 15% GelMA = 30 kPa [40]), adding nanodroplets do not seem to influence negatively on the stiffness of the network of GelMA, that is why deformation profile are relatively the same for both hydrogels. In the case of GelMA-Nanoliposomes, we observed that the deformation is much higher at equal strength showing a softer property. It is noted that the force required for a displacement of about 0.1 mm is equal to 13 N, this deformation can be due to the mechanical action of the nanoliposomes on the GelMA network. Nanoliposomes are soft particles anchored in the GelMA scaffold, which explains the same deformation of GelMA-Nanoliposomes needs less external force.

### 2.8. Viscoelastic Measurements

Dynamic shear oscillation measurement at small strain was used to characterize the viscoelastic properties and related differences in the molecular structure of functionalized hydrogels based on GelMA.

#### 2.8.1. Amplitude Sweep Test

**Figure 7:**
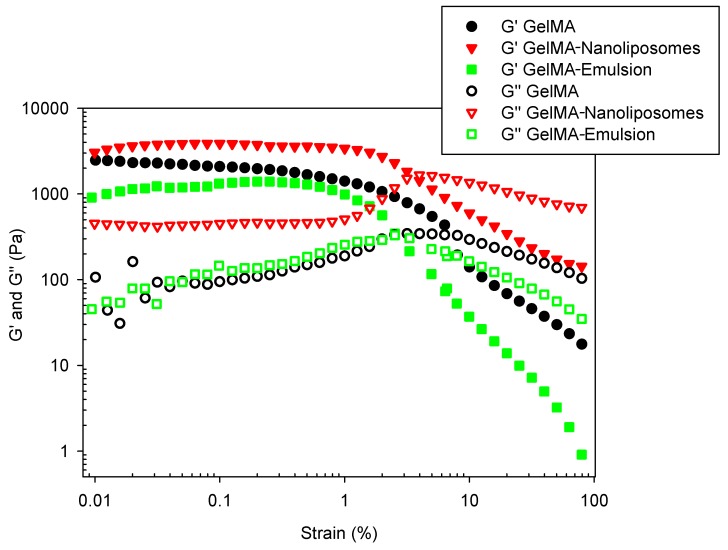
Amplitude sweeps showing storage moduli (G′) and loss moduli (G″) of the hydrogels performed over the whole strain range at a frequency of 1 Hz.

To determine the linear viscoelastic region (LVER), an amplitude sweep test was performed over strain range (from 0.01 to 100%). The graph (Figure 7) gives an indication of stability. While sample structure is maintained (between 0.1% and 1%), the complex modulus is constant. When the applied stress becomes too high (beyond 1%), decomposition of the internal structure occurs, and the modulus decreases. All the samples have the same LVER. The length of the LVER is a measure of stability that is why frequency sweep test must be performed in the LVER. The amplitude sweeps of the hydrogels also showed that all of them had a G′/G″ >> 1, typical of a stronger hydrogel character.

#### 2.8.2. Frequency Sweeps Test

**Figure 8:**
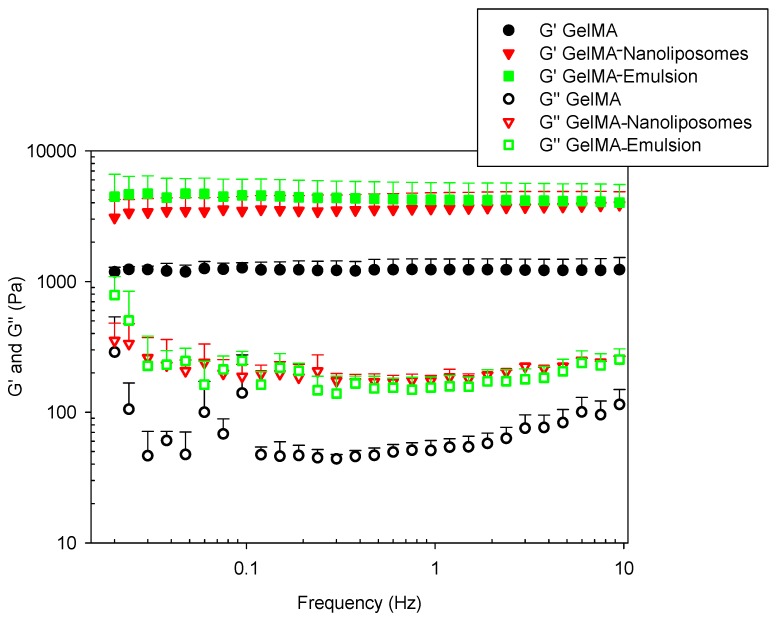
Frequency sweep test of hydrogels. (■, ●, and ▼) elastic modulus (G′) and (□, ○ and ∇) viscous modulus (G″) at a shear strain of 0.1%.

Rheological frequency sweep tests were performed on three-dimensional (discs) GelMA gel. The elastic (G′) and viscous (G″) moduli of hydrogels were investigated by dynamic mechanical analyses. The frequency dependence of these moduli was reported in Figure 8.

It is known that physical gelation results from a thermo-reversible conformation change from the triple helix to individual polypeptide coils at above approximately 40 °C. Upon cooling below 35 °C, the random coils join locally and associate into helix, which grows interconnect and forms larger domains until the whole volume is percolated [41]. Chemical cross-linking results through photo-polymerization of vinyl groups after UV initiation [27].

The first observation was that in all hydrogels, elastic modulus values G′ exhibits a pronounced plateau in the frequency range investigated. G′ values were superior to viscous modulus values G″, confirming that the pure GelMA hydrogel and functionalized GelMA hydrogels have a predominantly elastic rather than viscous character. This criterion discriminates viscoelastic solids behavior such as hydrogels from viscous liquids. Thus, the deformation energy is recovered in the elastic stretching of chemical bonds [42]. The constant values of functionalized hydrogels’ storage modulus with frequency sweep indicated the absence of relaxation processes, which may be explained by a stability of the intermolecular junctions. There has been no release of nanoparticles that would have resulted in a change in G′ value.

The high degree of methacrylation and the presence of strong chemical cross-links allow an elevated stability despite gel oscillatory in the frequency range (0.01 to 10 Hz). In this case, the contribution of physical cross-links cannot explain the high values because the experiment was done at 37 °C.

The incorporation of nanoliposomes and nanodroplets results in a significant increase of both G′ and G″. Considering that GelMA provides a big part of the hydrogel mechanical stability, the incorporation of nanoliposomes in the network walls and the adsorption of droplets on GelMA scaffold improve the elastic component of the resulting hydrogels.

## 3. Materials and Methods

### 3.1. Material

Gelatin (type A, 300 bloom from porcine skin), methacrylic anhydride (MA), photoinitiator (PI), 2-hydroxy-4′-(2-hydroxyethoxy)-2-methylpropiophenone and phosphate buffered saline tablets (PBS) were purchased from Sigma-Aldrich (Chemie, Stuttgart, Germany). Rapeseed lecithin was acquired from Solae Europe SA society (Geneva, Switzerland). Rapeseed oil (vegetable oil, Lesieur, Asnière-sur-Seine, France) was purchased from a local supermarket (Nancy, France).

### 3.2. Methacrylated Gelatin Synthesis

Methacrylated gelatin was synthesized as described previously [26]. Briefly, type A porcine skin gelatin was mixed at 10% (*w*/*v*) into phosphate buffered saline solution at 60 °C and stirred until fully dissolved. Then, 8 mL of MA was added very slowly and dropwise under stirring into the first solution. After 3 h, the reaction was stopped following a 1:5 dilution using warm phosphate buffered saline at 50 °C and allowed to react for 1 h. The mixture was then dialyzed for one week at 40 °C against distilled water using a dialysis membrane (Spectro/Por molecular porous membrane tubing, MWCO 12–14,000, SpectrumLabs, Inc., Rancho Dominguez, CA, USA) to remove salts and methacrylic acid. The solution was finally lyophilized for one week to generate white porous foam and stored at −20 °C until further use.

### 3.3. Nanoliposomes Preparation

We prepared 5% nanoliposomes solution by adding 5 g of rapeseed lecithin to 95 mL of water. The suspension was mixed for 5–6 h under agitation at inert atmosphere (nitrogen) and a temperature of 40 °C. Then, the lecithin solution was passed one time through high pressure homogenizer microfluidizer (Microfluidics LM 20, Newton, MA, USA). The lecithin solution was fed into the microfluidizer through a 300 mL glass reservoir (pressurized by an intensifier pump) and was passed through the homogenization unit for one pass at a constant pressure of 500 bar (50 MPa). Liposome samples were stored in glass bottle in the dark at 4 °C after preparation.

### 3.4. Nanoemulsion Preparation

A stable rapeseed oil in water emulsion was prepared according to GENIALIS patent (N149668A1) by a low frequency/high frequency (LF/HF) ultrasound method. 10% (*w*/*w*) oil/water dispersion was homogenized 3 min at 12,000 rpm then sonicated at low frequency at 20 kHz for 3 min (30 s on, 30 s off, amplitude 40%) in an ice bath. The dispersion was placed in a device composed of a thermostated reactor (4 °C) in which was located three piezoelectric transducers, a peristaltic pump, and a stirring modulus for 4.

The oil ratio in emulsion was calculated at the end of emulsification process by measuring the difference between oil initially put in emulsion and non-emulsified oil remaining at emulsion surface as follows:

(1) $$EC=\frac{\left(IV-FV\right)}{EV}$$

where *EC* is the emulsification capacity, *IV* the initial oil volume, *FV* the floating oil volume and *EV* the emulsion volume.

### 3.5. Hydrogels Preparation

#### 3.5.1. GelMA Hydrogel

Freeze-dried GelMA macromer was mixed at 15% (*w*/*v*) into PBS solution containing 1% (*w*/*v*) 2-hydroxy-1-(4-(hydroxyethoxy) phenyl)-2-methyl-1-propanone as a photoinitiator at 80 °C until fully dissolved.

One mL of 15% GelMA solution was poured on a specific silicone mold with the controlled dimensions (ø = 2 cm, h = 0.2 cm) and then exposed to UV light (360–480 nm) for 240 s. The PI absorbs the UV light and transforms the solution onto gel. The obtained gel was then washed with PBS before use.

#### 3.5.2. Nanoliposomes/GelMA Hydrogel

One mL of GelMA/nanoliposomes mixture (0.5 mL 30% GelMA + 0.5 mL 3% nanoliposomes + 1% PI) was poured on specific silicone mold and exposed to UV light (360–480 nm) for 240 s. The solution was transformed to gel by the action of PI reaction. The obtained gel (15% GelMA:1.5% nanoliposomes:0.5% PI) was then washed with PBS before use.

#### 3.5.3. Nanoemulsion/GelMA Hydrogel

One mL of GelMA/nanoemulsion mixture (0.5 mL 30% GelMA + 0.5 mL 3% emulsion + 1% PI) was poured on specific silicone mold and exposed to UV light for 240 s. The obtained gel (15% GelMA:1.5% nanoemulsions:0.5% PI) was then washed with PBS before use.

### 3.6. Size Measurement

The size distribution (mean diameter and polydispersity index) of the liposome dispersions was measured by dynamic light scattering (DLS) using a Malvern ZetasizerNano ZS (Malvern Instruments Ltd., Malvern, UK). Prior to measuring size, the samples were diluted (nanoliposomes: 1/400 and nanoemulsion 1/100) into a distilled water ultra-filtrate which measures the mass distribution of particle size. Measurements were made at 37 °C with a fixed angle of 173°. Sizes quoted are the *z*-average mean (dz) for the liposomal hydrodynamic diameter (nm). Measurements were made in triplicate.

### 3.7. Zeta Potential Measurements

Nanoliposomes and nanoemulsions Zeta potentials were measured with ZetasizerNano ZS (Malvern Instruments Ltd., Malvern, UK) using dynamic light scattering (DLS). The determined potential is an important parameter to analyze the effect of the nanoparticles in suspension. The samples were diluted (as previously) and introduced into disposable capillary cells equipped with gold electrodes designed to afford maximum zeta potential measurement capability. All measurements were carried out at 37 °C.

### 3.8. Scanning Electron Microscopy

The surface morphology of the different hydrogels was characterized by Quanta 200 high-resolution scanning electron microscope low vacuum mode (FEI-Japan, Tokyo, Japan). The use of “low vacuum” mode presents powerful tools for the observation of the surface topography of biological materials without sputter-coated. It also preserves the delicate samples from the electron beam damaging. The maximal resolution attained, employing an electron beam spot size of 3, could be lower than 5 nanometers. The analysis was executed by the use of a large field detector (LFD). The squared shaped samples with dimensions 9 × 9 mm² were inserted and maintained in a holder inside the SEM chamber and the tests were performed at laboratory temperature of 25 °C with a relative humidity of 50%. A partial vacuum was created within the chamber, and the air was evacuated using a pump, which provides a regular pressure of 60 mbar. The images were taken from a distance of 10.0 mm with an acceleration voltage of 10.00 kV. The pictures were provided utilizing software “xT microscope server.”

### 3.9. Fourier-Transform Infrared Spectroscopy

Fourier-transform infrared (FTIR) spectra of freeze-dried nanoliposomes, emulsion and hydrogels were recorded with a Tensor 27 mid-FTIR Bruker spectrometer (Bruker, Karlsruhe, Germany) equipped with a diamond ATR (Attenuated Total Reflectance) module and a DTGS (Deuterated-Triglycine Sulfate) detector. Between 4000 and 400 cm^−1^ at 4 cm^−1^ resolutions, 128 scans were used for both reference and samples. Spectral manipulations were then performed using OPUS software (Bruker, Karlsruhe, Germany). Raw absorbance spectra were smoothed using a nine-points smoothing function. After elastic baseline correction using 200 points, H_2_O/CO_2_ correction was then applied. Then, spectra were centered and normalized. All tests were run in triplicate.

### 3.10. Swelling Behavior Study

For determining the water retention capability of the prepared hydrogels, immediately following hydrogel formation three replicas (discs of ø = 2 cm, h = 0.2 cm) of each hydrogel were dipped in deionized water or phosphate buffer saline solution (PBS–pH 7.4) at 37 °C. The swelling ratios of hydrogels were measured after 20 min, 40 min, 60 min, 2 h, 4 h, 8 h, 24 h and 48 h (discs were dried with absorbent paper to remove the residual liquid before each weighing and dipped in water or PBS after).

The swelling ratio (*SR*) was calculated using the following equation:

(2) $$SR=\frac{\left(W_{f}-W_{i}\right)}{W_{i}}$$

where *W_f_* is the final weight and *W_i_* is the initial weight.

### 3.11. Characterization of Hydrogel Degradation with Collagenase

PBS pre-incubated discs of GelMA, GelMA-Nanoliposomes, and GelMA-Emulsion hydrogels were added to collagenase type II (crude collagenase from Clostridium histolyticum, reference number C-6885, Sigma-Aldrich) in PBS containing 2 µg/mL (0.5 U/mL) of enzyme. Samples were then placed in an oven at 37 °C. At the indicated time points, hydrogel samples were removed, wiped, and weighed. Mass loss was determined by normalizing sample weight measurements at time zero.

### 3.12. Mechanical Stability

To investigate mechanical stability of nanofunctionalized GelMA hydrogels, the three different systems were compressed individually between two parallel plates. A rotational rheometer Malvern kinexux (Malvern Instruments Ltd., Malvern, UK) with a plate-and-plate (20 mm) geometry was used. A force gap test used to compress the discs from 1.50 to 0.10 mm with a linear compression speed of 10 µm/s [43]. The gap and the normal force being imposed were measured simultaneously at the upper plate. At least three replicates were considered for each type of hydrogel.

### 3.13. Viscoelastic Measurements

Dynamic viscoelastic measurements were carried out using a Kinexus pro rheometer (Malvern Instruments Ltd., Malvern, UK) equipped with a plate-and-plate geometry (20 mm). A dynamic frequency sweep test from 0.02 to 30 Hz was performed to determine the dynamic storage (G′) and viscous (G″) modulus, at a strain rate of 0.1% confirmed to be in the linear viscoelastic range (LVER) for each type of hydrogel by a prior strain amplitude sweep (strain: from 0.01 to 100% at a frequency of 1.00 Hz).

During the rheological experiments, the temperature was maintained at 37 °C and the measuring system was covered with a humidity chamber to minimize water evaporation. Three different hydrogel disks were tested for each type of hydrogel with the same experimental settings and average values are presented.

## 4. Conclusions

In this report, we developed two approaches to synthesize functionalized GelMA hydrogels for cosmetic applications. Physicochemical and mechanical proprieties were characterized.

Both high shear fluid and LF/HF sonication processes have enabled us to make stable negatively charged nanoparticles, which can be used as nanovehicles to encapsulate and transport active molecules. FTIR results indicated the presence of a small amount of nanoparticles that does not disturb the polymer organization. No interactions have been revealed between the nanoparticles and GelMA scaffold.

SEM images demonstrated that functionalized hydrogels were porous and that they could serve as niches where eukaryotic cells can grow, interact, and benefit from a regular intake of active substances conveyed by nanoliposomes.

The mechanical properties of functionalized hydrogels investigated by small amplitude oscillatory shear rheology showed greater resistance to shear and twist and therefore better stability after functionalization.

## Figures and Tables

**Table 1:** Mean particle size (nm), polydispersity index (PDI), and zeta potential (mV) of the rapeseed nanoliposomes and nanodroplets of rapeseed oil emulsion.

Solutions	Particle Size (nm)	Polydispersity Index	Zeta Potential (mV)
Nanoliposomes	169.7 ± 2	0.360 ± 0.03	−43.7 ± 1.6
Nanoemulsion	237.9 ± 7	0.393 ± 0.01	−48.8 ± 0.6
